# Chronicity of Uncorrected Hyponatremia and Clinical Outcomes in Older Patients Undergoing Hip Fracture Repair

**DOI:** 10.3389/fmed.2020.00263

**Published:** 2020-06-30

**Authors:** Juan Carlos Ayus, Nora Fuentes, Alan S. Go, Steven G. Achinger, Michael L. Moritz, Sagar U. Nigwekar, Sushrut S. Waikar, Armando Luis Negri

**Affiliations:** ^1^Hospital Italiano de Buenos Aires, Buenos Aires, Argentina; ^2^Department of Nephrology, University of California–Irvine, Irvine, CA, United States; ^3^Renal Consultants, Houston, TX, United States; ^4^Internal Medicine Research Unit, Italian Hospital of Buenos Aires, Buenos Aires, Argentina; ^5^Research Department, Private Community Hospital, Mar del Plata, Argentina; ^6^Research Group of Non-communicable Chronic Diseases, Higher School of Medicine, National University of Mar del Plata, Mar del Plata, Argentina; ^7^Division of Research, Kaiser Permanente Northern California, Oakland, CA, United States; ^8^Departments of Medicine (Nephrology), Epidemiology and Biostatistics, University of California, San Francisco, San Francisco, CA, United States; ^9^Departments of Medicine (Nephrology), Health Research and Policy, Stanford University, Palo Alto, CA, United States; ^10^Department of Nephrology, Watson Clinic, Lakeland, FL, United States; ^11^Division of Nephrology, Department of Pediatrics, UPMC Children's Hospital of Pittsburgh, The University of Pittsburgh School of Medicine, Pittsburgh, PA, United States; ^12^Division of Nephrology, Department of Medicine, Massachusetts General Hospital, Boston, MA, United States; ^13^Nephrology Section, Boston University Medical Center, Boston, MA, United States; ^14^Instituto de Investigaciones Metabólicas, Universidad del Salvador, Buenos Aires, Argentina

**Keywords:** hyponatremia, hip fracture, osteopoenia/osteoporosis, elderly patients, mortality

## Abstract

**Background:** Chronic hyponatremia is a risk factor for hip fracture but remains uncorrected in most patients. This study evaluated if preoperative chronicity of uncorrected hyponatremia influences outcomes after hip fracture repair.

**Materials and Methods:** Evaluated were older patients hospitalized for hip fracture repair between 2007 and 2012 with plasma sodium measured at admission and ≥1 preadmission outpatient measurement. Patients were classified as being normonatremic (NN; plasma sodium 135–145 mmol/L), chronic prolonged hyponatremia (CPH; ≥2 consecutive plasma sodium values <135 mmol/L over >90 days), or recent hyponatremia (one plasma sodium <135 mmol/L within 30 days before admission with previously normal plasma sodium). Length of hospital stay, in-hospital death, post-operative complications, 30-day readmission, and long-term mortality were the evaluated outcomes. Multivariable Cox regression was used to evaluate the association of hyponatremia status with outcomes.

**Results:** Among 1,571 eligible patients, 76.7% were NN, 14% had CPH, and 9.1% had RH. Compared with NN patients, CHN patients were older and had more prior heart failure, alcoholism, and anticonvulsant drug use. In multivariable analyses, neither CPH or RH was associated with hospital length of stay, in-hospital or 30-day death, or 30-day readmission, while RH was associated with post-operative sepsis [adjusted odds ratio (aOR) 1.84, 95% CI: 1.01–3.35). Only CPH was independently associated with long-term all-cause death (OR 1.53, 95% CI: 1.12–2.09).

**Conclusions:** Hyponatremia affects nearly 25% of patients undergoing hip fracture repair. Preoperative chronic untreated hyponatremia is associated with increased post-operative mortality following surgical repair of a hip fracture in older patients. Future studies should evaluate if correction of hyponatremia could decrease long-term mortality after hip fracture repair.

## Introduction

Acute hyponatremia and chronic hyponatremia (plasma sodium <135 mmol/L) are known to be associated with significant morbidity and mortality (1–4). Hyponatremia is common among older adults, affecting an estimated 10% of free-living adults aged ≥65 years and up to 20% of persons aged ≥60 years living in a nursing home (5). Hyponatremia has also been associated with bone abnormalities, (6) and we recently observed that prolonged chronic hyponatremia in older adults is independently associated with an increased risk of experiencing a hip fracture (7). Hyponatremia is also linked to excess all-cause mortality in hospitalized patients with and without hip fracture, (7–9) yet the majority of patients with mild hyponatremia are discharged from the hospital while still remaining hyponatremic (10).

Several studies (11–14) have analyzed outcomes after surgical repair of a hip fracture in those with hyponatremia on admission or after surgery and have suggested excess short- and long-term mortality as well as prolonged length of hospital stay associated with hyponatremia. Existing studies did not differentiate between preadmission chronic hyponatremia compared with more recent-onset hyponatremia that existed before surgery. What is less clear is how uncorrected hyponatremia modifies this association.

To address some key knowledge gaps, this study examined a cohort of adults hospitalized for hip fracture repair to evaluate the influence of uncorrected preoperative hyponatremia and its chronicity on the risk of post-operative complications, length of stay, hospital readmission, and long-term mortality. It was hypothesized that both uncorrected chronic hyponatremia and recent-onset preoperative hyponatremia are independently associated with increased risks of adverse outcomes and excess mortality.

## Materials and Methods

### Setting, Sampling Period, and Data Sources

The source population included beneficiaries of the Hospital Italiano de Buenos Aires Care Program (HIBACP), a prepaid health maintenance organization in the city of Buenos Aires, Argentina, that currently provides comprehensive care for 150,000 persons. All study patients received treatment at the Hospital Italiano de Buenos Aires between January 1, 2007, and December 1, 2012. The data sources for the study included information extracted from the institution's electronic health record (EHR) system (including data from a central laboratory), the institutional registry for sepsis (IRS, www.ClinicalTrials.gov Identifier: NCT01403935), and the institutional registry for thromboembolic disease (IRTD www.ClinicalTrials.gov Identifier: NCT01372514). All patients' health care information in the institution is stored in a Clinical Data Repository (CDR), which has been operating for more than 10 years. The CDR has mirrored databases with de-identified information to ensure the privacy and confidentiality of the data. To enable the secondary analysis, the CDR was used as an information source. This repository stores clinical documents from different services such as test results, images, clinical notes, drug prescriptions, pharmacy dispensations, outpatients visits, ER visits, in-hospital care, among other examples. Therefore, the information in this cohort was collected from high-quality secondary health care database systems of the Hospital network, integrated into the Electronic Medical Record (EMR) with a relational base model.

The study was approved by the Hospital Italiano de Buenos Aires' institutional review board and was carried out in compliance with the principles outlined in the Declaration of Helsinki. A waiver of informed consent was obtained due to the nature of the study.

### Study Sample and Classification of Normonatremia and Hyponatremia Status

A retrospective cohort study was conducted including all patients older than 18 years of age who underwent surgery for traumatic hip fracture and had ≥1 plasma sodium measured preadmission, at admission, and ≥1 measurement before surgery. All patients included in the study had preadmission plasma sodium determinations. Patients with a traumatic hip fracture were identified using relevant Systematized Nomenclature of Medicine-Clinical Terms (SNOMED-CT) codes from emergency department or hospital primary discharge diagnosis information found in the EHR (7). Each patient's primary treating inpatient physician reviewed relevant medical records, including radiographic imaging and operative reports, in order to confirm that there was a solitary traumatic hip fracture and to exclude pathologic hip fractures, such as bone metastases, or multiple traumatic fractures.

All plasma sodium concentration measurements were performed using the ion-selective electrode method (normal range 135–145 mmol/L). Plasma sodium levels were corrected for plasma glucose using the following formula: corrected plasma sodium (mEq/L) = measured plasma sodium (mEq/L) + 0.016 ^*^ (plasma glucose [mg/dL]−100). Using available information from a central laboratory as of the date of hospital admission, patients were classified as being normonatremic or hyponatremic, with further subclassification of hyponatremia. Using available preadmission and admission plasma sodium data, patients with plasma sodium fluctuations above or below 135 mmol/L were excluded from the analytic sample to avoid misclassification of natremia status, and those with persistent values >145 mmol/L consistent with hypernatremia were also excluded. Among the remaining patients, to be considered normonatremic, patients had to have all preadmission and admission plasma sodium values between 135 and 145 mmol/L. Patients with hyponatremia were categorized as having either chronic prolonged hyponatremia (defined as having ≥2 consecutive plasma sodium values <135 mmol/L over a period of more than 90 days before admission) or recent hyponatremia (defined as having one plasma sodium determination <135 mmol/L within 30 days before admission with all previous available values being between 135 and 145 mmol/L). Hyponatremia was not corrected, and patients remained hyponatremic during the entire follow-up period after surgery.

### Covariates

Preadmission patient characteristics that could be associated with increased in-hospital morality included age, gender, body mass index, ischemic heart disease, chronic heart failure, dementia, liver failure, gait disorders, and chronic kidney disease (CKD, defined as an estimated glomerular filtration rate <60 ml/min/1.73 m^2^, using the Modification of Diet in Renal Disease Study equation). Clinical risk factors for fractures present in the FRAX algorithm were included. The fracture risk algorithm (FRAX) was developed by the World Health Organization to calculate the 10-year probability of a hip fracture and the 10-year probability of any major osteoporotic fracture (defined as clinical spine, hip, forearm, or humerus fracture) in a given patient. These calculations account for femoral neck bone mineral density (BMD) and other clinical risk factors, as follows: age, sex, body mass index, personal history of fracture, use of oral glucocorticoid therapy, secondary osteoporosis (e.g., coexistence of rheumatoid arthritis), parental history of hip fracture, current smoking status, and alcohol intake (three or more drinks per day). The World Health Organization risk assessment tool is available at http://www.shef.ac.uk/FRAX.

Preadmission medication use that may be associated with hyponatremia included chronic use of antidepressant medications (selective serotonin reuptake inhibitors, tricyclic antidepressants), anticonvulsants, thiazide diuretics, and proton pump inhibitors and corticosteroid use based on information from admission notes and medication lists.

### Follow-Up and Outcomes

Follow-up occurred through December 31, 2012. Outcomes of interest included both in-hospital and post-discharge outcomes. Post-operative complications (within 30 days after hip surgery) that were evaluated included the development of acute myocardial infarction, heart failure, significant atrial or ventricular arrhythmias, stroke, venous thromboembolism (deep venous thrombosis or pulmonary embolism), and sepsis. In-hospital mortality, length of hospital stay (in days), hospital readmission for any cause within 30 days after discharge, and death from any cause during follow-up were evaluated using comprehensive data from the EHR.

### Statistical Analysis

Statistical analyses were performed using SPSS 19.0 statistical software, and a two-sided *P* <0.05 was considered significant in all analyses. Continuous variables were reported as either mean and standard deviation (SD) or median and interquartile range (IQR) if the variable was non-normally distributed. Categorical variables were reported as frequencies and proportions. Pairwise comparisons were made between groups for continuous variables using a *t-t*est or the Kruskal–Wallis test, as appropriate, and for categorical covariables using a chi-square test or Fisher's exact test. The proportion of patients experiencing in-hospital death and readmission within 30 days post-discharge across groups was compared using chi-square tests. Index hospitalization length of stay was compared across groups using a *t-*test. Incidence of all-cause death was compared across groups using Kaplan–Meier survival curves and a log-rank test.

To evaluate the independent association between natremia status and clinical outcomes, a propensity score was generated to predict the probability of presenting with any hyponatremia by considering as candidate variables the baseline patient characteristics and medications shown in Table 1. The final propensity score logistic regression model had a c statistic of 0.72.

**Table 1:** Baseline characteristic of hip fracture patients with normonatremia, chronic prolonged hyponatremia, and recent hyponatremia at admission.

		**Normonatremia (*n =* 1,205)**	**Chronic prolonged hyponatremia (*n =* 222)**	***P* (vs. normonatremia)**	**Recent hyponatremia (*n =* 144)**	***P* (vs. normonatremia)**
**Baseline characteristics**	Median (IQR) age (year)	82 (77–86)	86 (81–89)	<0.01	83 (78–87)	0.73
	Women	961 (79.8%)	183 (82.4%)	0.35	119 (82.6%)	0.41
	Median (IQR) plasma sodium (mmol/L)	138 (137–140)	131 (129–133)	<0.01	133 (131–134)	<0.001
	Median (IQR) body mass index (kg/m^2^)	26 (23–29)	25 (22–28)	0.023	25 (22–27)	0.04
	Diagnosed dementia	16 (1.3%)	4 (1.8%)	0.58	4 (2.8%)	0.17
**Risk factors associated with hyponatremia**	Prior heart failure	166 (13.8%)	63 (28.4%)	<0.01	20 (13.4%)	0.97
	Chronic kidney disease	407 (33.8%)	92 (41.4%)	0.028	44 (30.6%)	0.43
	Liver failure	9 (0.7%)	2 (0.5%)	0.80	0	0.29
	Antidepressant use	128 (10.6%)	27 (12.2%)	0.49	12 (8.3%)	0.39
	Diuretic use	37 (3.1%)	4 (1.8%)	0.29	5 (3.5%)	0.79
	Anticonvulsant agent use	103 (8.5%)	16 (7.2%)	0.50	4 (2.8%)	0.01
**FRAX**^®^ **clinical risk factors associated with hip fracture**	Osteoporosis	565 (46.9%)	116 (52.3%)	0.14	61 (42.4%)	0.30
	Rheumatoid arthritis	8 (0.7%)	3 (0.8%)	0.28	0	0.32
	Alcoholism	13 (1.1%)	6 (2.7%)	0.05	4 (2.8%)	0.08
	Smoking	183 (15.2%)	35 (15.8%)	0.82	21 (14.6%)	0.84
	Other fractures	885 (73.4%)	167 (75.2%)	0.58	100 (69.4%)	0.30
	Steroid use	51 (4.2%)	11 (5%)	0.62	2 (1.4%)	0.09

A multivariable logistic regression was performed for the dichotomous outcomes of in-hospital death and readmission for any cause within 30 days post-discharge. A multivariable linear regression was performed for index hospitalization length of stay. A multivariable Cox proportional hazard model was performed for the outcome of time to death from any cause. All multivariable models were adjusted for the propensity score for presenting with hyponatremia as a continuous variable in addition to directly adjusting for age, gender, body mass index, preadmission diagnosed dementia, prior heart failure, preexisting CKD, and risk factors for hip fracture, given that these variables may have differential prognostic information on the outcomes of interest. For the outcome of death from any cause, an additional model was conducted that included post-operative complications which may mediate, at least in part, any observed association between hyponatremia and mortality. The follow-up period was up to 5 years.

## Results

### Cohort Assembly and Baseline Characteristics

**Figure 1:**
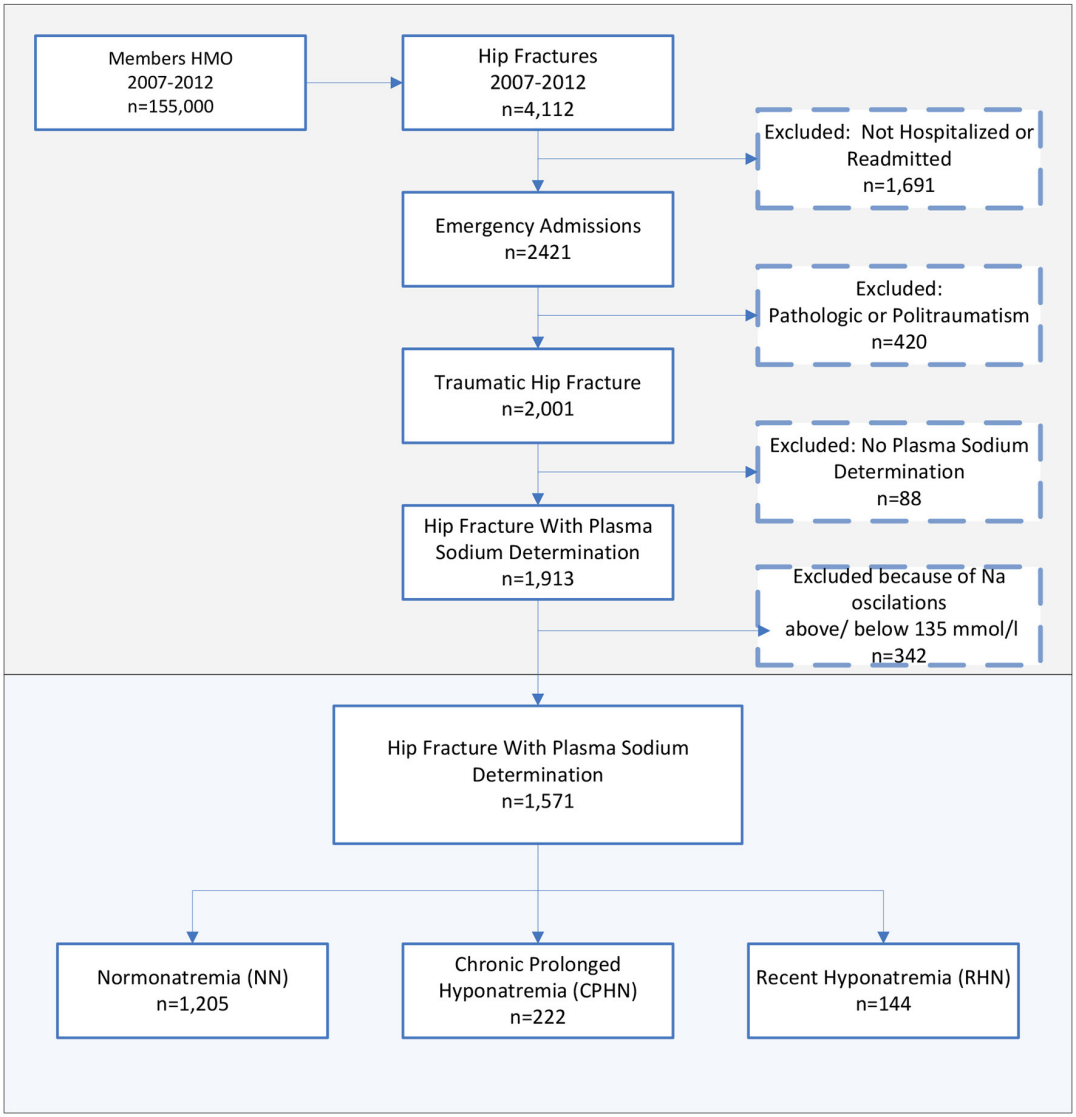
Cohort assembly of eligible patients hospitalized for hip fracture repair.

Between 2007 and 2012, 1,571 eligible adults were admitted for hip fracture repair, with 76.7% presenting with normonatremia (median plasma sodium 138 mmol/L), 14.2% presenting with chronic prolonged hyponatremia (median plasma sodium 131 mmol/L), and 9.1% presenting with recent hyponatremia (median plasma sodium 133 mmol/L) (Figure 1). Of note, among the 222 patients presenting with chronic prolonged hyponatremia, the estimated mean (SD) time with hyponatremia was 1,027 (649) days and the median (interquartile) time of hyponatremia was 932 days (445.25–1,484) before admission.

Compared with patients who were normonatremic, those presenting with chronic prolonged hyponatremia were older and were more likely to have prior heart failure, a history of alcoholism, preadmission anticonvulsant drug use, and a lower body mass index (Table 1). Compared with those who were normonatremic on admission, those presenting with recent hyponatremia were less likely to have preadmission anticonvulsant drug use and also had a lower body mass index (Table 1). Of note, there was no significant difference in the median time between admission and surgery in those who presented with normonatremia (1 day), chronic prolonged hyponatremia (1 day, *P* = 0.86), or recent hyponatremia (1 day, *P* = 0.78).

### Hyponatremia and Length of Stay and In-hospital Mortality

Compared to patients presenting with normonatremia (median length of stay = 7 days), the unadjusted median length of hospital stay was 8 days (*P* = 0.15) in those presenting with chronic prolonged hyponatremia and 8 days (*P* = 0.78) in those with recent hyponatremia. After adjustment for the propensity to present with any hyponatremia and other potentially confounding variables, no differences were found in length of stay with chronic prolonged hyponatremia (*P* = 0.97) nor in recent hyponatremia (*P* = 0.93) in the length of stay.

Crude risk of in-hospital death was 14.7% in patients presenting with normonatremia compared with 24.3% (*P* < 0.01) in those with chronic prolonged hyponatremia and 13.2% (*P* = 0.63) in patients with recent hyponatremia. In multivariable analyses, compared to those presenting with normonatremia, having chronic prolonged hyponatremia or recent hyponatremia was not significantly associated with in-hospital death (Table 2).

**Table 2:** In-hospital mortality and post-operative complications within 30 days by initial natremia status in patients undergoing hip fracture repair.

	**Normonatremia (*n =* 1,205)**	**Chronic prolonged hyponatremia (*n =* 222)**	**Adjusted odds ratio (95% CI)**	**Recent hyponatremia (*n =* 144)**	Adjusted^*^ odds ratio (95% CI)
In-hospital mortality	177 (14.7%)	54 (24.3%)	1.37 (0.92–1.93)	19 (13.2%)	1.29 (0.86–1.60)
Post-operative complication					
Sepsis	72 (6.0%)	21 (9.5%)	1.26 (0.74–2.14)	15 (10.4%)	1.84 (1.01–3.35)
Venous thromboembolic event	42 (3.5%)	9 (4.1%)	0.93 (0.43–1.99)	2 (1.4%)	0.40 (0.09–1.68)
Acute myocardial infarction	8 (0.7%)	1 (0.5%)	0.44 (0.05–3.73)	1 (0.7%)	1.08 (0.13–8.77)
Significant atrial or ventricular arrhythmia	20 (1.7%)	7 (3.2%)	1.52 (0.61–3.77)	3 (2.1%)	1.29 (0.37–4.42)
Stroke	10 (0.8%)	2 (0.9%)	0.89 (0.18–4.27)	0 (0%)	0
Heart failure	14 (1.2%)	6 (2.7%)	0.94 (0.33–2.67)	1 (0.7%)	0.88 (0.10–7.10)

* *Adjusted for propensity to present with hyponatremia, age, gender, and Charleston comorbidity score*.

### Hyponatremia and Post-operative Complications

The frequencies of post-operative complications within the first 30 days among groups are shown in Table 2. In multivariable analyses adjusting for the likelihood to present with any hyponatremia in addition to age, gender, and Charlson comorbidity score, chronic prolonged hyponatremia was not significantly associated with any of the post-operative complications, while recent hyponatremia was only associated with higher odds of post-operative sepsis (adjusted OR 1.84, 95% CI: 1.01–3.35) (Table 2).

### Hyponatremia and 30-Day Hospital Readmission and Long-Term Mortality

The crude risk of readmission for any cause at 30 days post-discharge was higher in patients presenting with chronic prolonged hyponatremia (18%, *P* = 0.04) but not recent hyponatremia (12.5%, *P* = 0.90) compared to patients with normonatremia (12.9%). After adjustment for potential confounders, there was no significant association with 30-day readmission for chronic prolonged hyponatremia (adjusted OR 1.29, 95% CI: 0.86–1.60) or recent hyponatremia (adjusted OR 0.93, 95% CI: 0.54–1.60).

**Figure 2:**
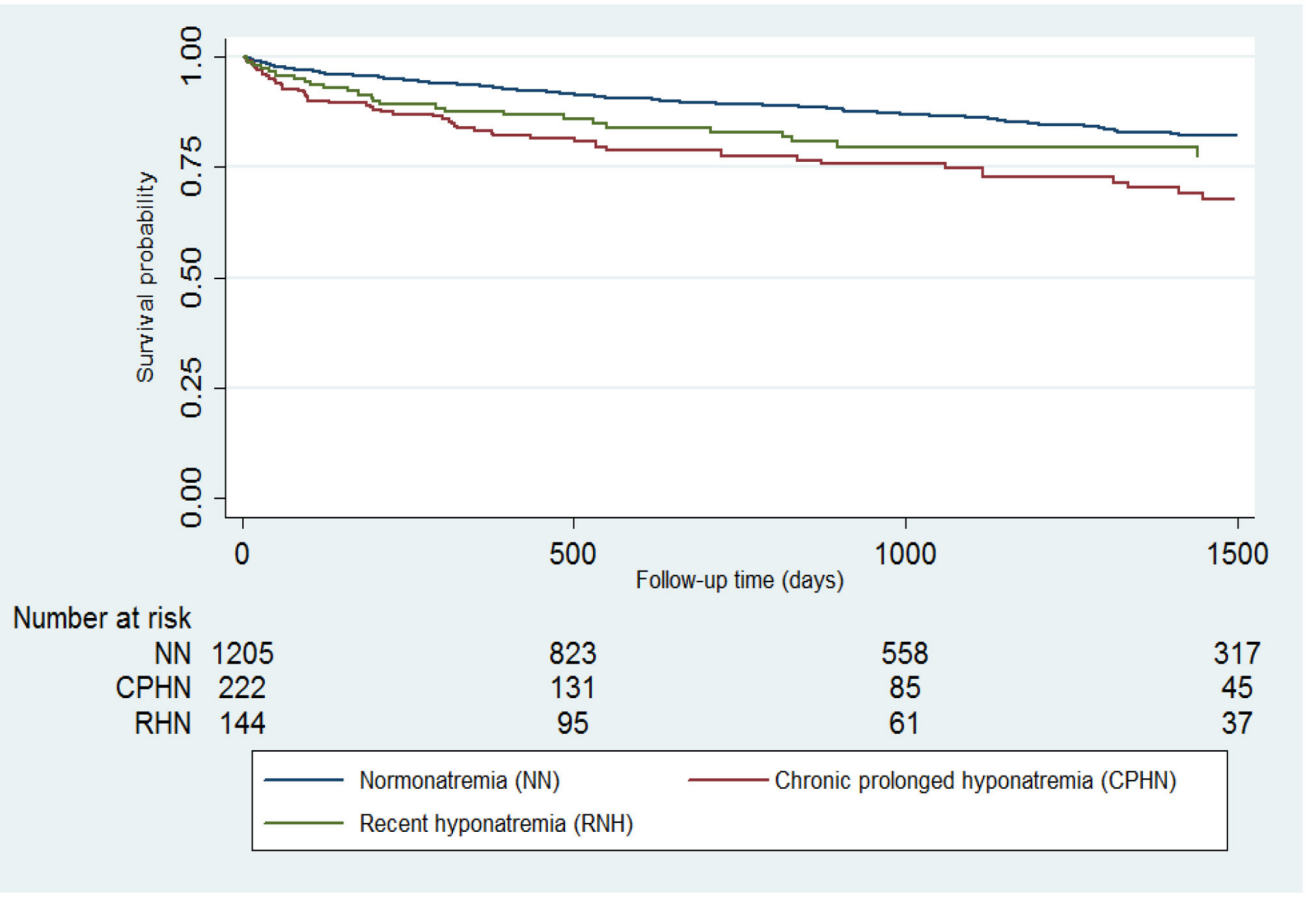
Long-term survival for patients undergoing hip fracture repair who presented with normonatremia, chronic prolonged hyponatremia, or recent hyponatremia.

Unadjusted survival was lower in patients presenting with chronic prolonged hyponatremia (*P* < 0.01) or recent hyponatremia (*P* = 0.046) (Figure 2). In Cox proportional hazards regression that adjusted for the likelihood of presenting with hyponatremia, as well as age, gender, Charlson comorbidity score, baseline medication use, and occurrence of post-operative complications, patients presenting chronic prolonged hyponatremia had a 53% significantly higher adjusted rate of death from any cause (adjusted hazard ratio 1.53, 95% CI: 1.12–2.09), while there was no significant association for recent hyponatremia (adjusted hazard ratio 1.29, 95% CI: 0.86–1.93). The mean Charlson comorbidity score in patients with 30-day in-hospital mortality or post-operative complications was 4 ± 2 SD.

## Discussion

Among older patients undergoing hip fracture repair, uncorrected chronic prolonged hyponatremia (mean plasma sodium of 131 mmol/L) on admission was independently associated with higher long-term all-cause mortality compared with presenting with normonatremia, while there was no significant adjusted difference in those presenting with recent hyponatremia. In contrast, neither chronic prolonged hyponatremia or recent hyponatremia was significantly associated with prolonged length of stay or in-hospital mortality during the index admission, or 30-day readmission for any cause, after accounting for a wide range of potential confounders, while recent hyponatremia was associated with higher adjusted odds post-operative sepsis.

Our findings support and extend previous studies that have reported that chronic hyponatremia is associated with excess mortality risk in various conditions (10, 15–18). This growing body of evidence highlights the potential systemic negative implications of chronic hyponatremia and raises the question about whether the central nervous system is the main, and perhaps only, end-organ target of systemic hyponatremia (19). Several studies have reported that hyponatremia is highly prevalent in patients undergoing surgical repair of a traumatic hip fracture, (13, 14, 20, 21) and this study similarly found that nearly one in four patients (23%) had hyponatremia at the time of hip fracture repair. While these studies have suggested excess short- and long-term mortality in those with hyponatremia, none characterized the duration of hyponatremia and its association with outcomes following hip fracture surgery. The major finding of our study is that post-discharge all-cause mortality was significantly higher in the group who had uncorrected chronic prolonged hyponatremia prior to hip fracture repair but not for recent-onset hyponatremia. Our results support that the chronicity of hyponatremia not only is important as a risk factor for developing a hip fracture, as we have shown previously, (7) but also is linked to an increased long-term risk of death after hip fracture repair as compared to older patients with shorter exposure to hyponatremia prior to hip fracture or to patients with normonatremia.

Chronic hyponatremia may have clinical consequences not fully appreciated until recently. In a rodent model of chronic hyponatremia, sustained hyponatremia for 18 weeks exacerbates multiple manifestations of senescence such as development of osteopenia, hypogonadism, decreased body fat, sarcopenia, and cardiomyopathy (19). If chronic prolonged hyponatremia could produce these conditions in human subjects, this could increase frailty in an aged population that may promote an excess risk of death. Emerging data indicate that hyponatremia may also be a risk factor for infection due to impaired function of interleukin (IL)-17 producing helper T-cells that play a key role in host immunity and breakdown of microbial target function due to cellular edema of mucosal membranes (22–25). Sepsis was the most frequent complication after traumatic hip fracture repair in this study (Table 2). Hyponatremia during the preoperative period has been recently associated with an increase in subsequent perioperative complications, such as wound infection and pneumonia (26). The syndrome of inappropriate anti-diuretic hormone (SIADH) is associated with excess mortality after discharge from hospital (27) and has been associated with an increased risk of death after pneumonia (28). In patients with SIADH who died within the first year, malignancy appeared to be the most common cause (25.4%), followed by infection (23.8%). Hyponatremia is an independent predictor of higher risk for infection-related hospitalization in chronic hemodialysis patients, and infectious complications may partially account for the increased mortality observed in the hyponatremic population with end-stage renal disease (ESRD) (29). Pretransplant hyponatremia (plasma sodium ≤ 130 mEq/L) was significantly associated with a higher adjusted risk for post-operative sepsis among 134 patients who underwent living-related liver transplantation (30). Not all studies have found that hyponatremia is associated with infection-related death though, (31) so additional research is needed to delineate the potential infectious and non-infectious pathways that may explain the excess post-operative mortality associated with preoperative chronic prolonged hyponatremia.

A strength of this study is that it included a large number of hip fracture patients who were systematically followed within a healthcare delivery system in which the duration of hyponatremia prior to surgery could be characterized and subsequent long-term mortality for patients with untreated sustained hyponatremia compared with recent hyponatremia or normonatremia. All the patients with plasma sodium fluctuations above or below 135 were excluded from the population studied to avoid misclassification of natremia status. We also leveraged clinically rich EHR data on a range of relevant possible preoperative confounding factors and comprehensive capture of post-operative complications and long-term follow-up for death.

This study also has several limitations. As an observational study of “real-world” practice, the study is susceptible to residual confounding as it relied on information documented in an EHR to identify potential confounders, and there may be misclassification or missing data. Information on over-the-counter medications was unavailable, and the specific cause(s) of death that occurred during follow-up could not be ascertained. The cause of death can be difficult to determine even if death certificate information is available due to misclassification (32, 33). Results from this study may not be fully generalizable to all populations given the limited racial/ethnic diversity and the source population based in Argentina. The study also did not evaluate whether chronic prolonged hyponatremia is a modifiable risk factor for all-cause death.

In summary, preoperative chronic untreated hyponatremia is associated with increased post-operative mortality following surgical repair of a hip fracture in older patients. This supports the concept that chronic hyponatremia is not a benign condition, and future interventional studies are needed to determine if correction of hyponatremia decreases long-term mortality in this high-risk group.

## Data Availability Statement

The datasets generated for this study are available on request to the corresponding author.

## Author Contributions

JA and AN conceived of the study. NF and AG were involved in the statistical design and analysis. JA, AN, MM, SA, SN, and SW were involved in the study design, analysis, and editing of the manuscript. All authors contributed to the article and approved the submitted version.

## Conflict of Interest

The authors declare that the research was conducted in the absence of any commercial or financial relationships that could be construed as a potential conflict of interest.
